# Generating fair, reliable, and accurate neuropsychological test norms for people with HIV in a low- or middle-income country

**DOI:** 10.1007/s13365-024-01235-6

**Published:** 2024-12-30

**Authors:** H. Gouse, K. G. F. Thomas, C. J. Masson, M. Henry, J. A. Joska, L. A. Cysique, S. Ling, X. Ye, J. Liu, R. N. Robbins

**Affiliations:** 1 Department of Psychiatry and Mental Health, University of Cape Town, Cape Town, South Africa; 2 ACSENT Laboratory, Department of Psychology, University of Cape Town, Cape Town, South Africa; 3 Centre for Higher Education Development, University of Cape Town, Cape Town, South Africa; 4 Department of Psychology, the University of New South Wales, Sydney, Australia; 5 Mailman School of Public Health, Columbia University, New York, NY USA; 6 Harvard T.H. Chan School of Public Health, Boston, MA USA; 7 HIV Center for Clinical and Behavioral Science, New York State Psychiatric Institute and Columbia University, New York, NY USA; 8 New York State Psychiatric Institute, New York, USA

**Keywords:** Cross-cultural assessment, HIV-associated neurocognitive impairment, HIV-associated neurocognitive disorder, Low- and middle-income country (LMIC), Normative data, Regression-based norms

## Abstract

Effective neuropsychological assessment of people with HIV (PWH) in low- and middle-income countries (LMICs) is hampered by the unavailability of adequate test norms. We aimed to: (1) develop demographically-corrected (regression-based) South African (SA) normative data for an HIV appropriate neuropsychological test battery for Xhosa home-language speakers; (2) compare the utility of those norms to that of (i) internal standardization norms and (ii) US test publisher norms; and (3) determine the criterion validity of the newly-developed norms. 114 controls and 102 demographically comparable Xhosa home-language people living with HIV completed a well-establised, standard HIV neuropsychological test battery assessing seven cognitive domains. Using a common performance metric (*z*-score), we compared control and PWH test performance and examined the extent to which the three different normative datasets embedded demographic effects e.g., education. Using internal standardization norms, analyses detected medium-sized correlations of overall test performance with age and education. Correlations were fully corrected for by the newly-developed demographically-corrected norms. Using demographically-corrected norms, PWH performed significantly more poorly than controls in five cognitive domains, whereas using internal standardization norms and test-publisher norms, PWH performed significantly more poorly than controls in one and two domains, respectively. Demographically-corrected norms estimated 43.1% of PWH were cognitively impaired; these estimates were 22.5% using test-publisher norms and 19.6% using internal standardization norms. Demographically-corrected SA norms were more sensitive to cognitive impairment in PWH than the other sets of norms. Expansion of this regression-based method to create population-appropriate norms will benefit research and clinical practice in LMICs.

**Supplementary Information:**

The online version contains supplementary material available at 10.1007/s13365-024-01235-6.

## Introduction

South Africa is a culturally, ethnically, linguistically, socioeconomically, and educationally diverse country that, globally, contains the highest number of people with HIV (PWH; Shisana et al. 2014; UNAIDS 2019). Among South African PWH, there is a high prevalence of HIV-associated neurocognitive impairment (NCI; 30–50%; Joska et al. 2011). Significant impairment in activities of daily living (e.g., less effective use of the Internet, poorer driving performance, incomplete medication adherence) has been associated with even mild NCI in PWH (Gouse et al. 2021; Marcotte et al. 2004; Ripamonti & Clerici 2021; Woods et al. 2017; Woods & Sullivan 2019).

Despite the fact that many major research studies and clinical centers use a collection of standard tests to assess performance in cognitive domains typically affected by HIV, few locally appropriate norms are available for those tests (Grant 2008; Watts & Shuttleworth-Edwards 2016). The aim of this study was to develop regression-based norms that can be used to assess neuropsychological test performance among PWH in South Africa (Deist et al. 2023).

Globally, there is a great demand for fair and accurate neuropsychological assessment of diverse populations. Neuropsychological testing in low- and middle-income countries (LMICs), where HIV is most prevalent, faces the triple challenge of: (1) highly diverse (e.g., ethnically, linguistically, economically, educationally) patient populations, (2) a lack of tests adapted and normed for these diverse populations, and (3) few resources to validate and norm tests for local populations (Nyamayaro et al. 2019b; Robbins et al. 2014). Many neuropsychologists in LMICs therefore opt to use test instruments and associated normative databases originating in high-income countries (HICs) such as the United States (Nyamayaro et al. 2019a), despite the fact that a central component of fair neuropsychological assessment is a set of valid, reliable, and standardized tests with norms appropriate for the person being assessed (American Education Research Association 2014).

Reliance on HIC-developed norms is almost always inappropriate for LMIC-based neuropsychological assessment (Cysique et al. 2014; Fernández & Abe 2018). Mismatches between local and standardization samples across key demographic factors (e.g., educational background, socioeconomic status [SES], ethnicity, culture, and language), as well as the greater exposure of LMIC individuals to community violence, poverty, malnutrition, institutionalized discrimination, and structural inequalities (e.g., in wealth and healthcare) are clear contraindications for using publisher-provided HIC-based norms (Brickman et al. 2006; Fernández & Abe 2018; Manly et al. 2004, 2002; Manly & Echemendia 2007).

This situation, alongside general global inequalities in healthcare resources, has forced clinicians and researchers in LMICs to search for pragmatic solutions to generating test norms that maintain high standards of accuracy and fairness (American Education Research Association 2014; International Test Commission 2019). Although population-based norms frequently allow those standards to be met, they are not particularly pragmatic because they require very large sample sizes (*N* of many 1000s), especially when recruitment is required across key strata of diverse populations (e.g., age, sex, level of education, quality of education, geographical location, home language/ethnicity, SES; Bridges & Holler 2007; Crawford & Garthwaite 2008; International Test Commission 2019; Lenhard et al. 2018; Manly & Echemendia 2007; Miller et al. 2015; Oosterhuis et al. 2016). Many LMICs simply do not have the resources to carry out such an undertaking.

One solution, then, is to collect performance estimates from samples of control participants and to generate norms based on those uncorrected estimates; in the current study, these are referred to as *internal standardization norms.* These sets of normative data, which are often derived from data collected to match experimental samples in research studies, tend to have small sample sizes drawn from a specific subgroup of the population because they aim to capture core variables representing that subgroup (Laher et al. 2019; Strauss et al. 2006).

Although the use of control-derived internal standardization norms is appropriate and effective under some conditions, there are important cautions to consider before taking that approach. One, of course, is the issue of sample size. Relatedly, these normative datasets may not represent the wider population particularly accurately; they certainly do not control as effectively for population-wide sociodemographic differences as good population-based norms do. Further, one must be careful to avoid sampling issues (e.g., an over-reliance on convenience sampling as might arise when recruitment from a healthcare setting results in a medically at-risk sample) that might bias test performance and that might lead to the construction of a group that does not approximate a true cross-section of the healthy population (Mitrushina et al. 2005; Nyamayaro et al. 2019b). The distribution of control-derived internal standardization normative data is also often characterized by large confidence intervals around performance estimates (scaled, standardized, or *z*-scores; Casaletto & Heaton 2017).

To address these potential pitfalls, test developers and psychometric research groups are using regression-based norms with increasing frequency (Casaletto et al. 2015; Damasceno et al. 2018; Deist et al. 2023; Heaton et al. 2004; Lenhard et al. 2018; Maroof 2012). There are several advantages to using regression-based techniques to generate normative data (Cysique et al. 2011; Fastenau 1998; Heaton et al. 1991; Testa et al. 2009). For instance, influential variables such as age and education can be treated as continuous and can be incorporated simultaneously into multivariate models that predict test performance after sociodemographic correction (Temkin et al. 1999). Moreover, regression-based norms require a 2.5 to 5.5 times smaller sample size than population-based norms to obtain equally precise estimates of impairment (Oosterhuis et al. 2016). Provided that regression-based norms meet statistical assumptions (e.g., normality, homogeneity of variances, linearity, and independence), the method may provide a feasible solution to the lack of locally appropriate norms in resource-limited LMICs (Burggraaff et al. 2017; Cysique et al. 2011; Duff & Ramezani 2015; Gott et al. 2017; Heaton et al. 2015; Parmenter et al. 2010).

The purpose of this study was to (1) develop a set of regression-based norms – that is to say, demographically-corrected South African (SA) norms – for a battery of neuropsychological tests widely used when assessing PWH, and (2) compare the utility of these newly-developed norms to that of two other types of normative data (US test-publisher norms and internal standardization norms) in appraising neuropsychological test performance and rates of neurocognitive impairment among PWH in South Africa. We note here that our purpose was not to characterize HIV-associated neurocognitive impairment; that characterization is the subject of numerous previous investigations, including some focusing specifically on the South African population of PWH (see, e.g., Heaton et al. 2010; Heaton et al. 2015; Joska et al. 2011).

Of particular interest in our analyses were (a) the extent to which demographic influences on test performance remained detectable when using the set of regression-based norms as opposed to the other sets of norms, (b) how estimates of performance differences between controls and PWH varied with use of the different normative datasets, and (c) how estimates of neurocognitive impairment among PWH varies with use of the different normative datasets.

## Method

The data reported here were collected between March 2014 and November 2016. The Human Research Ethics Committee of the Faculty of Health Sciences at the University of Cape Town approved the study's protocols and procedures.

### Participants

All participants were recruited from a low-income community (Khayelitsha, Cape Town), similar to that of other South African communities where there are typically high rates of HIV. Khayelitsha has an unemployment rate of 38.8%. Almost three-quarters of its residents (73.8%) earn less than ZAR3200 (± US$213) per month, 54.7% live in informal dwellings, and only 4.9% have ≥ 12 years of education (Strategic Development Information GIS 2013). Regarding education, it is likely that most participants attended schools that were relatively poorly resourced (e.g., with high student-to-teacher ratios, small or no libraries, and no computer facilities).

#### Control sample:

One hundred and fourteen HIV-negative adults were recruited from two public health clinics and the surrounding communities. We used stratified sampling to ensure that men and women were equally represented and that there were relatively equal numbers of people in different age- and education-based sub-groups (see Table 1).

**Table 1:** Healthy Control Participants: Sociodemographic Characteristics Stratified by Sex, Age, and Education (*N* = 114)

	Sex (*n*, %)			Education (yrs; *n*, %)			
Age Range (yrs)	Male	Female	Total *n*	8–9	10–11	12–13	Total *n*
18–24	14 (51.85)	13 (48.14)	27	8 (29.63)	9 (33.33)	10 (37.04)	27
25–34	15 (50.00)	15 (50.00)	30	9 (30.00)	11 (36.67)	10 (33.33)	30
35–44	14 (50.00)	14 (50.00)	28	8 (28.57)	10 (35.71)	10 (35.71)	28
45–64	16 (55.17)	13 (44.83)	29	11 (37.93)	9 (31.03)	8 (27.59)	28
Total	59	55	114	36	39	38	113

One person in the age bracket of 45–54 years had 7 years of education

Inclusion criteria were being (a) HIV-negative as confirmed by an enzyme-linked immunosorbent assay (ELISA), (b) aged 18 years or older, (c) English- or Xhosa-speaking, (d) capable of consent, and (e) willing to complete a comprehensive neuropsychological test battery. Participants also had to report having no history of any of the following: diabetes mellitus, head injury with loss of consciousness > 30 min or resulting in hospitalization, coma, psychiatric disorder, congenital disorder, or central nervous system disorder (e.g., epilepsy). We also excluded those with clinically significant depressive symptoms (i.e., score > 17 on the Beck Depression Inventory-II; Beck et al. 1996), a positive screening test for alcohol use disorder (as measured by the Alcohol Use Disorders Identification Test [AUDIT], cut-off score ≥ 8; Bohn et al. 1995), or a positive urine toxicology screen for marijuana, methamphetamine, or opioids.

#### People with HIV:

One hundred and two adults with HIV were recruited from the same two public health clinics. They had recently completed a randomized controlled trial (RCT) of an adherence intervention for PWH initiating antiretroviral therapy (ART; Gouse et al. 2017; Robbins et al. 2018). They were enrolled into this study after completing their last RCT study visit (i.e., at 12 months post-ART initiation).

Study eligibility criteria for this group were identical to those described above for controls except that these participants were required to be HIV-positive and were not excluded if they had diabetes.

### Measures and procedure

All participants were screened for eligibility, provided written informed consent, and were compensated the equivalent of US$40 after completing all study procedures. We used standard versions of all tests described below except the Hopkins Verbal Learning Test-Revised (HVLT-R); in that case, we used a culturally adapted version, frequently used in South African clinical settings, that combines Forms 1 and 4 of the original (Nyamayaro et al. 2019b; Scott et al. 2018). All tests were forward- and back-translated into Xhosa, with final versions settled by group consensus, to ensure linguistic validation.

The test administrator and scorer was trained, supervised, and monitored by a US-based clinical psychologist (RNR) and a South African-based clinical neuropsychologist (HG).

*Neuropsychological Assessment.* A bilingual neuropsychology technician administered a comprehensive neuropsychological test battery using a mix of English and Xhosa instructions. HVLT-R stimuli were presented in the language of the participant’s preference (in the large majority of cases this was Xhosa) (Scott et al. 2018). Digit Span stimuli were always presented in English and category fluency answers were accepted in both languages.

The battery contained 14 standardized tests that assessed performance across seven cognitive domains, using these specific outcome variables: (1) Executive Functioning: Color Trails Test 2 (CTT2) completion time and the Wisconsin Card Sorting Test (WCST) total correct; (2) Learning: HVLT-R total learning and the Brief Visuospatial Memory Test-Revised (BVMT-R) total learning; (3) Memory: HVLT-R delayed recall total and BVMT-R delayed recall total; (4) Language: category fluency (total number of animals / total number of fruits and vegetables named in 1 min); (5) Attention/Working Memory: Wechsler Memory Scale-Third Edition (WMS-III) Spatial Span total raw score and Wechsler Adult Intelligence Scale-Third Edition (WAIS-III) Digit Span total raw score; (6) Processing Speed: Trail Making Test Part A (TMT-A) completion time, CTT1 completion time, WAIS-III Digit Symbol Coding total score, WAIS-III Symbol Search total raw score; and (7) Motor Function: Grooved Pegboard Test (GPT) completion time.

Table S1 lists the sources of test-publisher norms for each test. We used normative data from the published test manual for each test except for the category fluency tests, for which there are no test-publisher normative data.

### Statistical analyses

We used R Studio (version 1.2) and SPSS (version 26.0) to conduct the analyses, with the threshold for statistical significance set at *p* < 0.05 (unless noted otherwise) and effect size estimates interpreted following conventional guidelines (Cohen 1988).

Three sets of *z-*scores were generated for each participant for each test. Demographically corrected SA norms (i.e., regression-based norms) were derived using standard procedures described by Testa et al. (2009). Specifically, we (a) transformed all raw scores from the control sample to scaled scores (*M* = 10, *SD* = 3) using the cumulative frequency distribution of each test; (b) conducted a multiple regression analysis for each scaled score with sex, age, age-centered squared, education (in years), and education-centered squared as predictors; (c) created a *z*-score for each outcome variable by dividing the residuals (actual scaled score minus predicted scaled score) by the standard deviation of the control group residuals (i.e., the standard error of the estimate); (d) transformed PWH raw test scores to scaled scores using the control group scaled scores; and (e) used the multiple regression equation results to compute demographically predicted *z*-scores for PWH and controls.

Internal standardization norms (i.e., control-derived norms based) were calculated using data from the control sample’s test performance. Specifically, we used the mean and standard deviation from each test outcome variable to create individual *z*-scores.

Test publisher norms (i.e., US-publisher normative data correcting for age, gender, and/or education) were calculated by (a) using the sources listed in Table S1 to extract scaled scores for all individual raw test scores and (b) converting those scaled scores to *z*-scores (i.e., a scaled score of 10 is equal to a *z*-score of 0). Although demographically-adjusted norms created outside of SA are available for some tests in our battery, these norms are less readily available to SA clinicians than norms published in test manuals, and we therefore chose to use US publisher norms.

After creating each of the three sets of normative data as describe above, we created three independent sets of domain *z*-scores (i.e., one set of for demographically corrected SA norms, one set for internal standardization norms, and one set for test publisher norms) by summing the mean z-scores of all the test outcome variables in a particular cognitive domain and dividing that sum by the number of outcomes in the domain. Then, we took the average of those individual *z*-scores to create a global *z*-score (i.e., a score that reflected average cognitive performance across the set of tests).

With all the neuropsychological test data processed and the sets of *z*-scores for each normative dataset in hand, we proceeded to the main inferential analyses. First, Pearson correlation coefficients assessed the degree of influence key sociodemographic variables (age, education, sex) had on overall cognitive performance in the control sample as measured by each normative dataset separately (i.e., global *z*-score for the control sample estimated by demographically corrected SA norms, by internal standardization norms, and by test publisher norms).

Second, a series of independent-sample *t*-tests (or Mann–Whitney *U* tests, where appropriate) assessed the magnitude of between-group (controls versus PWH) differences on cognitive outcome variables (*z*-scores describing global performance as well performance on each individual test and within each discrete cognitive domain) as estimated separately by each normative dataset.

Third, we set out to determine a form of criterion validity for each normative dataset by generating, for each of them, estimated rates of neurocognitive impairment within the PWH group. Following the proposition that approximately 15% of people in the normal population meet criteria for NCI on neurocognitive testing (see Carey et al. 2004; Taylor & Heaton 2001), for each norming method we searched each domain individually for the cut-off *z*-score that allowed for approximately 15% of controls participants to be classified as impaired (i.e., a *z*-score less than the identified threshold) in 2 or more domains. We then set the cut-off for impairment in each cognitive domain as close as possible to 15% for the controls, thus attempting to balance Type I and Type II errors. Once we had completed those preliminary steps, we created two different outcome variables for the purposes of subsequent analyses of rates of neurocognitive impairment within the PWH group: (a) Overall Neurocognitive Impairment, with the threshold for impairment defined as domain *z*-score lower than the identified cut-off in two or more cognitive domains, and (b) Global Deficit Score (GDS; based on global rating algorithms used in HIV, see Carey et al. 2004; Heaton et al. 1994), with the threshold for impairment defined as a score > 0.5. For the latter, we used all tests except Fruit and Vegetables, for which there were no suitable published norms. Fisher’s Exact tests assessed, for each normative dataset separately, the magnitude of between-group differences for each cognitive domain and for Overall Neurocognitive Impairment.

Fourth, McNemar tests of dependent proportions and follow-up pairwise Cochran’s *Q* tests assessed, for each discrete cognitive domain and for each of the control and PWH groups separately, the magnitude of between-norms differences in the proportion of participants with Overall NCI.

Fifth, within each of the control and PWH groups, Mann–Whitney *U* (for continuous variables) and chi-square (for categorical variables) tests compared demographic factors between those classified as cognitively-impaired and cognitively-normal as determined by (a) the test publisher norms, (b) the internal standardization norms, and (c) the demographically corrected SA norms.

## Results

### Sample characteristics

All participants were Xhosa home-language speakers. As Table 2 shows, the control and PWH groups were quite well matched in age (control range = 18–64 years; PWH range = 19–56 years) and number of years of completed education (control range = 7–13 years; PWH range = 8–14 years). There were, however, proportionally more women in the PWH group than in the control group: Whereas the latter had a relatively even sex distribution, more than 80% of the former group’s participants were women. This disproportionate representation of women in the PWH group accurately reflects the South African clinical context (Zuma et al. 2022).

**Table 2:** Between-group comparisons on demographic variables and HIV biological data

	Control (SD)	HIV (SD)	*p*
Age (years)	35.44 (11.95)	33.31 (7.46)	.381^a^
Education (years)	10.54 (1.43)	11.25 (1.99)	.003
BDI-II total score	8.14 (5.07)	4.10 (7.41)	< .001
Gender (female)	50.9%	81.4%	< .001
HIV			
CD4 cell count^b^		501.31 (287.41)	

^a^Mann-Whitney *U*test performed.^b^CD4: Cluster of differentiation 4; CD4 cell count available for 88 participants. *SD* Standard deviation

HIV-disease characteristics are reported in Table 2. CD4 count ranged from 47 – 1654.

Table S2 provides descriptive statistics for performance by the control group on the complete set of neuropsychological tests. We used those data to create the set of internal standardization SA norms (see Table S3). We used the final equations from the regression models for each neuropsychological test (see Tables S4.1–S4.7) to derive the demographically-corrected SA norms.

### Bivariate correlations: Overall cognitive performance and sociodemographic variables

As Table 3 shows, analyses detected no significant associations between the global *z*-score as estimated by the demographically corrected SA norms and any of the three key sociodemographic variables under consideration (sex, age, education). In contrast, the global *z*-score as estimated by internal standardization norms was significantly negatively correlated with both age and education (*p* < 0.001 in both cases). Similarly, the global *z*-score as estimated by test publisher norms was significantly negatively correlated with age (*p* < 0.001) and significantly positively correlated with education (*p* < 0.001).

**Table 3:** Pearson correlation between demographics and overall z-scores for each normative standard (N = 114)

	Gender	Age	Education
SA corrected norms overall *z*-score	-.027	-.037	.014
Internal standardization norms overall *z*-score	-.050	-.465**	-.456**
US publisher norms overall *z*-score	.077	-.296**	.411**

**Correlation is significant at the 0.001 level (2-tailed)

### Neuropsychological test performance: Between-group comparisons using different normative standards

Table 4 displays descriptive statistics (means and standard deviations) for three sets of *z*-scores representing the test performance of the control and PWH groups, with each set calculated using one of the three different normative methodologies. Of note in this Table is that, when using the test publisher norms, all *z*-scores for both controls and PWH were negative. This pattern of data suggests that, when using this set of norms, the average test-taker in both groups (regardless of whether they present with a clinical condition or not) will score poorly. Evidently, then, some form of normative adjustment must be made in order to reflect cognitive capabilities more accurately.

**Table 4:** Neuropsychological test performance: Between-group comparisons within each method of deriving normative data (*N* = 216)

			Methodology									
	US Norms				Internal-standardization Norms				Demographically-corrected SA Norms			
	HC	PWH		Effect	HC	PWH		Effect	HC	PWH		Effect
Domain / Test/ Outcome Variable	(*n* = 114)	(*n* = 102)	*p*	size	(*n* = 114)	(*n* = 102)	*p*	size	(*n* = 114)	(*n* = 102)	*p*	size
Motor Function												
Grooved Pegboard Test												
Dominant hand	−0.50 (2.50)	−1.66 (2.56)	.001**	**0.46**	0.00 (1.00)	−0.26 (0.80)	.038*	**0.29** 0.10	0.00 (1.00)	−0.87 (1.24)	< .001***	**0.78** **0**
Nondominant hand	−0.88 (4.16)	−1.88 (2.31)	.033*	**0.29**	0.00 (1.00)	−0.08 (0.41)	.438	0.10	0.00 (1.00)	−0.76 (1.28)	< .001***	**0.67**
Processing Speed												
TMT-A	−1.69 (1.73)	−2.76 (2.07)	< .001***	**0.56** **0.60**	0.00 (1.00)	−0.62 (1.20)	< .001***	**0.56** **0.330.33**	0.00 (1.00)	−1.04 (1.16)	< .001***^a^	**0.43**
CTT1	−0.65 (1.11)	−1.30 (1.04)	< .001***	**0.60**	0.00 (1.00)	−0.33 (1.00)	.017*	**0.33** **0.29** **0.29**	0.00 (1.00)	−0.78 (0.97)	< .001***	**0.79**
WAIS-III ymbol Search	−1.47 (0.70)	−1.21 (0.59)	.005**	**0.40**	0.00 (1.00)	−0.28 (0.90)	.034*	**0.29**	0.00 (1.00)	−0.41 (1.14)	.005**	**0.38**
WAIS-III Digit Symbol	−1.62 (0.49)	−1.79 (0.51)	.017*	**0.34**	0.00 (1.00)	−0.33 (1.01)	.017*	**0.33**	0.00 (1.00)	−1.03 (1.23)	< .001***	**0.92**
Attention/Working Memory												
WMS-III Spatial Span	−1.73 (0.70)	−1.69 (0.73)	.671	0.06	0.00 (1.00)	0.05 (0.97)	.731	0.05 0.47	0.00 (1.00)	−0.03 (1.09)	.851	0.03
WAIS-III Digit Span	−1.13 (0.61)	−1.37 (0.49)	.004**^a^	**0.20**	0.00 (1.00)	−0.43 (0.79)	< .001***^a^	**0.47**	0.00 (1.00)	−0.63 (0.95)	< .001***	**0.65**
Language												
Category Fluency												
Animals	−1.40 (0.85)	−1.49 (0.70)	.376	0.11	0.00 (1.00)	−0.06 (0.87)	.644	0.06	0.00 (1.00)	−0.36 (1.17)	.015*	**0.33**
Fruits and Vegetables^b^	––	––	––	–-	0.00 (1.00)	0.18 (0.87)	.155	0.19	0.00 (1.00)	−0.35 (1.00)	.009**	**0.35**
Learning												
HVLT-R Total Learning	−0.89 (1.02)	−1.25 (0.80)	.003^**a^	**0.21**	0.00 (1.00)	−0.40 (0.83)	.002**	**0.43**	0.00 (1.00)	−0.65 (0.83)	< .001***	**0.70**
BVMT-R Total Learning	−2.47 (0.74)	−2.57 (0.66)	.318	0.14	0.00 (1.00)	−0.16 (0.98)	.238	0.16	0.00 (1.00)	−0.47 (1.09)	< .001***	**0.45**
Memory												
HVLT-R Delayed Recall Total	−0.90 (1.09)	−1.44 (1.06)	< .001***	**0.50**	0.00 (1.00)	−0.53 (0.99)	< .001***	**0.53**	0.00 (1.00)	−1.01 (1.18)	< .001***	**0.93**
BVMT-R Delayed Recall Total	−2.30 (0.90)	−2.25 (1.03)	.729	0.05	0.00 (1.00)	−0.04 (1.15)	.775	0.04	0.00 (1.00)	−0.33 (1.29)	.035*	**0.29**
Executive Function												
CTT2	−0.61 (1.18)	−0.94 (1.13)	.039*	**0.29**	0.00 (1.00)	−0.14 (1.00)	.312	0.14	0.00 (1.00)	−0.68 (1.07)	< .001***	**0.66**
WCST (Total Correct)^c^	−3.39 (1.30)^c^	−3.30 (1.40)	.597	0.10	0.00 (1.00)^c^	0.07 (1.15)	.403	0.07	0.00 (1.00)^c^	−0.04 (1.30)	.785	0.04

Data presented are *M*(*SD*) for *z*-scores. *HC* Healthy Controls, *PWH* People Living with HIV, *TMT-A* Trail Making Test Part A, *CTT1* Color Trails Test 1, *WAIS-III* Wechsler Adult Intelligence Scale, Third Edition, *WMS-III* Wechsler Memory Scale, Third Edition, *HVLT-R* Hopkins Verbal Learning Test-Revised, *BVMT-R* Brief Visuospatial Memory Test-Revised, *CTT2* Color Trails Test 2, *WCST* Wisconsin Card Sorting Test

^a^Result from Mann–Whitney *U* test. ^b^Manualized norms not available. ^c^HC data based on *n* = 112 (two participants did not complete the test)

*p < .05. **p < .01. ***p < .001. All listed *p*-values are two-tailed. Statistically significant *p*-values are marked in boldface font

Table 4 also shows that, when performance was evaluated against test publisher *z*-scores, PWH scored significantly more poorly than controls on 9 of 16 test outcomes and significantly better on 1 (Symbol Search). When performance was evaluated against internal standardization norms, PWH scored significantly more poorly than controls on 8 tests. When performance was evaluated against demographically corrected SA norms, PWH scored significantly more poorly than controls on 14 tests.

### Rates of neurocognitive impairment: Between-group comparisons using different normative standards

Table 5 provides the *z*-score, for each cognitive domain, that allowed approximately 15% of control participants to be classified as displaying Overall Neurocognitive Impairment.

**Table 5:** Z-score cut-offs for each cognitive domain resulting in 15% of controls being impaired on two or more domains (*N* = 114)

	z-score cut off		
Domain/Outcome variable	US-publisher Norms	Internal-standardization Norms	Demographically-corrected SA Norms
Executive Function	−3.12	−1.24	−0.98
Motor Function^a^	−2.47	−0.55	−0.54
Processing Speed	−2.7	−1.41	−1.13
Attention/Working Memory	−2.16	−1.17	−1.34
Language	–-^b^	−1.11	−1.10
Learning	−2.65	−1.12	−1.06
Memory	−2.67	−1.18	−1.17

^a^Data value for one participant was removed and winsorized. ^b^US-publisher norms were not available to calculate a domain score

Table 6 presents, for each norm-generating method separately, the proportion of participants in each group who displayed impaired performance (*z* ≤ the identified threshold) on each of the seven discrete cognitive domains. Using test publisher norms, significantly more PWH than controls were classified as impaired in the domains of Motor Function and Memory. Using internal standardization norms, significantly more PWH than controls were classified as impaired in the domain of Motor Function. Using demographically corrected SA norms, significantly more PWH than controls were classified as impaired in the domains of motor function, processing speed, learning, memory, and executive function. Here, effect sizes ranged from small (Cramer’s *V* = 0.08 for the Learning domain) to medium (Cramer’s *V* = 0.31 for the Motor Function domain).

**Table 6:** Rates of Neurocognitive Impairment: Between-group Comparisons of Percentage of Impaired Participants Within Each Method of Deriving Normative Data (N = 216)

	Methodology											
	US-publisher Norms				Internal-standardization Norms				Demographically-corrected SA Norms			
	HC (%)	PWH (%)			HC (%)	PWH (%)			HC (%)	PWH (%)		
Domain / Outcome Variable	(*n* = 114)	(*n* = 102)	*p*	ESE	(*n* = 114)	(*n* = 102)	*p*	ESE	(*n* = 114)	(*n* = 102)	*p*	ESE
Motor Function	9.7	25.5	.001**	.21	7.0	16.7	.027*	.15	7.9	32.4	< .001***	.31
Processing Speed	7.9	7.8	.495	< 0.01	8.8	9.8	.794	.02	7.0	23.5	< .001***	.23
Attention/Working Memory	10.5	12.7	.306	.04	6.1	6.9	.830	.02	7.0	7.8	.817	.02
Language	––	––	––	––	7.0	3.9	.321	.07	7.9	12.7	.239	.08
Learning	9.7	5.9	.153	.07	7.9	12.7	.239	.08	7.9	22.5	.002**	.21
Memory	9.7	17.6	.043*	.12	7.9	15.7	.074	.12	7.9	30.4	< .001***	.29
Executive Function	9.7	15.7	.090	.09	8.8	6.9	.603	.04	7.0	16.7	.027*	.15
Overall NCI (average z-score)	10.5	26.5	.001*	.21	7.9	8.8	.805	.02	7.0	25.5	< 001***	.24
Overall NCI (≥ 2 domains) impaired)	15.8	22.5	.103	.09	14.0	19.6	.273	.08	15.8	43.1	< .001***	.30

Data presented are percent of participants with impaired performance (i.e., *z* < 7.5th percentile cut-off for Internal standardization and Demographically corrected SA Norms, and *z* < 10th percentile cut-off for US-publisher Norms) on the domain in question. *HC* Healthy Controls, *PWH* People Living with HIV, *ESE* effect size estimate (Cramer’s *V*), *NCI* Neurocognitive Impairment

*p < .05. **p < .01. ***p < .001. All listed *p*-values are one-tailed. Statistically significant *p*-values are marked in boldface font

Regarding the proportion of participants in each group displaying Overall Neurocognitive Impairment, the only significant between-group difference was detected for data from the demographically corrected SA norms (see Table 6). When estimating performance using those norms, 43.1% of PWH (in contrast to 15.8% of controls) were judged as impaired, a difference associated with a medium-sized effect (Cramer’s *V* = 0.30). Using test-publisher norms and internal standardization norms, the estimates of impairment were 22.5% and 19.6% respectively.

Regarding the proportion of participants in each group who delivered impaired performance as measured using the GDS, again the only significant between-group difference was detected for data from the demographically corrected SA norms. When estimating performance using those norms, 37.3% of PWH (in contrast to 14.9% of controls) were impaired, a difference associated with a medium-sized effect (Cramer’s *V* = 0.26).

### Proportion of participants with domain-based neurocognitive impairment identified by different normative standards: within-group comparisons

Within the control group, omnibus between-method comparisons detected no statistically significant differences (all *p*s > 0.072; see Table 7). Within the PWH group, however, the analyses detected statistically significant differences for all domain scores (all *p*s < 0.001 except Attention/Working Memory [*p* = 0.021]).

**Table 7:** Rates of Neurocognitive Impairment as Determined by US Norms vs Uncorrected SA Norms vs Demographically-corrected SA Norms: Within Group Follow-up Pairwise Comparisons for Each Group (N = 216)

	Main Effect		Pairwise Comparison		Pairwise Comparison		Pairwise Comparison	
	Cohran *Q* Test		Internal-standardization Norms vs Demographically-corrected SA Norms		US vs Internal-standardization Norms		US vs Demographically-corrected SA Norms	
	HC	PWH	HC	PWH	HC	PWH	HC	PWH
Domain / Outcome Variable	(*n* = 114)	(*n* = 102)	(*n* = 114)	(*n* = 102)	(*n* = 114)	(*n* = 102)	(*n* = 114)	(*n* = 102)
Motor Function	.662	< .001***	1.00	< .001***	1.00	.082	1.00	.259
Processing Speed	.717	< .001***	1.00	.001**	1.00	1.00	1.00	< .001***
Attention/Working Memory	.072	.021*	1.00	1.00	.091	.028*	.250	.091
Language^a^	–	–	1.00	.031*	–	–	–	–
Learning	. 717	< .001***	1.00	.049*	1.00	.278	1.00	< .001***
Memory	.670	< .001***	1.00	< .001***	1.00	1.00	1.00	.002**
Executive Function	.529	< .001***	1.00	.001**	1.00	.004**	.804	1.00
Overall NCI (average z-score)	.623	< .001***	1.00	< .001***	.549	< .001***	.607	1.00
Overall NCI (≥ 2 domains) impaired)	.827	< .001***	.791	< .001***	.791	.581	1.00	< .001***

Data presented are *p*-values associated with Cochran’s *Q* tests. *HC* Healthy Controls, *PWH * People Living with HIV, *TMT-A* Trail Making Test Part A, *CTT1* Color Trails Test 1, *WAIS-III* Wechsler Adult Intelligence Scale, Third Edition, *WMS-III* Wechsler Memory Scale, Third Edition, *HVLT-R* Hopkins Verbal Learning Test-Revised, *BVMT-R* Brief Visuospatial Memory Test-Revised, *DR* Delayed Recall trial, *CTT2* Color Trails Test 2, *WCST* Wisconsin Card Sorting Test

^a^Result from McNemar test

*p < .05. **p < .01. ***p < .001. All p-values were adjusted for multiple comparisons using Bonferroni correction for multiple tests. All listed *p*-values are two-tailed. Statistically significant *p*-values are marked in boldface font

Regarding the Language domains, omibus between method copmarisons could not be conducted for any analyses that involved US based norms as there were no US based norms for semantic fluency. Within the PWH group, pairwise comparisons between internal-standardization and demographically-corrected SA norms found a statistically significant differene (*p* = 0.031).

Table 7 also shows results from other follow-up pairwise comparisons (one norming method versus another) for data from the PWH group. In the comparison of demographically corrected SA norms against internal standardization norms, the former classified significantly more participants as impaired in the domains of motor function, processing speed, learning, memory, and executive function (i.e., in all domains except attention/working memory). In contrast, in the comparison of demographically corrected SA norms against test publisher norms the latter classified significantly more participants as impaired than in the domains of processing speed, learning, and memory. Finally, in the comparison of test publisher norms against internal standardization norms, the former classified significantly more participants as impaired in the domains of attention/working memory and executive function.

### Participants classified as cognitively impaired versus those classified as cognitively unimpaired: between-group comparisons on sociodemographic variables

As Table 8 shows, the only between-group differences that were detected by these analyses occurred when classification was by internal standardization norms. Specifically, using that normative dataset (a) control participants classified as cognitively impaired had significantly fewer years of education than those classified as cognitively unimpaired, (b) PWH participants classified as cognitively impaired had significantly fewer years of education than those classified as cognitively unimpaired, and (c) control participants classified as cognitively impaired were significantly older than those classified as cognitively unimpaired.

**Table 8:** Demographic Factors in Healthy Controls and People with HIV classified as cognitively normal vs. cognitively impaired using internal-standardization SA norms and demographically corrected norms (as classified by impairment in two or more domains

	US-publisher Norms				Internal-standardization Norms				Demographically-corrected SA Norms			
	Cognitively impaired (*n* = 18)	Cognitively normal *(n* = 96)	*z/χ*^*2*^	*p*	Cognitively impaired (*n* = 16)	Cognitively normal *(n* = 98)	*z/χ*^*2*^	*p*	Cognitively impaired (*n* = 18)	Cognitively normal (*n* = 96)	*z/χ*^*2*^	*p*
	Median (IQR)	Median (IQR)			Median (IQR)	Median (IQR)			Median (IQR)	Median (IQR)		
**HC**												
Age^a^	36.5 (21.5 – 44.8)	34 (25 – 47.8)	−0.09	0.926	44 (38.3 −54)	33 (24 – 44)	−3.20	0.001**	37 (24 – 46.3)	33.5 (25 – 44.8)	−0.40	0.686
Education^a^	10 (9 – 11)	11 (9 – 12)	−1.95	0.051	9 (8 – 10.8)	11 (9 – 12)	−3.60	< 0.001***	10 (9 – 11)	11 (9 – 12)	−1.50	0.134
Sex (male)^b^	9 (50%)	47 (49%)	0.01	0.935	9 (56.3%)	47 (48%)	0.38	0.539	8 (44.4%)	48 (50%)	0.19	0.665
**PWH**	(*n* = 23)	(*n* = 79)			(*n* = 20)	(*n* = 82)			(*n* = 44)	(*n* = 58)		
Age^a^	32 (26 – 37)	33 (28 – 38)	−0.57	0.572	35 (30 – 41)	32 (28 – 38)	−1.17	0.241	31 (26.3 – 37.8)	33 (28.8 – 38)	−1.09	0.276
Education^a^	11 (9 – 12)	12 (11 – 12)	−1.60	0.109	11 (8.3 – 12)	12 (11 – 12)	−2.83	0.005**	12 (11—13)	12 (10.8 – 12)	−1.33	0.184
Sex (male)^b^	5 (21.7%)	14 (17.7%)	0.19	0.663	6 (30%)	13 (15.9%)	2.12	0.145	5 (11.4%)	14 (24.1%)	2.69	0.101

^a^Mann-Whitney *U* tests performed. ^b^Chi-square tests performed. *HC* Healthy Controls, *PWH* People with HIV

*p < 0.05. **p < 0.01. ***p < 0.001. Statistically significant *p*-values are marked in boldface font

When classification was by test publisher norms or by demographically corrected SA norms, analyses detected no significant between-group (cognitively impaired versus unimpaired) differences in terms of age, education, or sex distribution, for either control or PWH participants.

## Discussion

We created a novel set of demographically corrected (regression-based) norms using data from a group of healthy South African adults (see 10.25375/uct.14718192). Our analyses of neuropsychological test data from control participants and people with HIV (PWH) showed that, compared to US-based test publisher norms and local internal standardization norms, this novel normative dataset (a) most effectively corrected for demographic (i.e., non-organic) effects on test performance, and (b) appeared to capture most accurately HIV-associated deficit patterns and overall prevalence of cognitive impairment. Hence, we argue that this set of demographically corrected SA norms can be used with confidence in South African NeuroHIV research and in patient samples with similar demographic profiles to our control group.

Our first major set of inferential analyses compared the test performance of PWH against that of a control group, using each of the three normative datasets in turn. The main finding here was that the demographically corrected SA norms appeared more sensitive to low performance. PWH performed significantly more poorly than controls on 14 of the 16 test outcome variables when performance was evaluated against demographically corrected SA norms, on 9 outcomes when evaluated against test publisher norms, and on 8 outcomes when evaluated against internal standardization norms. Generally speaking, the pattern of deficits detected by demographically corrected SA norms mirrored the known patterns of neurocognitive impairment observed in cART-era PWH (Heaton et al. 2010; Heaton et al. 2015). Of note is that only when evaluated against the internal standardization norms did PWH not perform more poorly on any executive function tests.

Our second major set of inferential analyses assessed, for each cognitive domain, data regarding the proportion of people with NCI identified by the three different normative standards. To implement these analyses, we first calculated (using the control data only, and for each cognitive domain separately) the *z*-score cut-off that characterized approximately 15% of the control group as impaired. Then we defined Overall Neurocognitive Impairment (for both controls and PWH participants) as impaired performance in two or more cognitive domains (see Table 6; Carey et al. 2004; Taylor & Heaton 2001). However, when evaluating Overall NCI against test publisher norms, almost all participants were classed as impaired – this then required us to calculate another set of *z*-scores based on thresholds below which approximately 15% of controls scored (see Table 5). For test publisher norms, domain-based cut-offs for impairment in the control group ranged from −3.12 ≤ *z* ≤ −2.16. For both the internal standardization norms and the demographically corrected SA norms, the *z*-score cut-offs were closer to the more realistic *z*-score of −1.0, suggesting that these sets of norms have better application in our context.

Consistent with our previous set of results, the demographically corrected SA norms showed greater sensitivity to impairment than did the other two sets of norms. When performance was evaluated against the demographically corrected SA norms, significantly more PWH than controls met the criterion for impairment on five of the seven domain scores. This contrasts to three domains for test publisher norms and one domain for internal standardization norms.

Using the cognitive domain score to establish a form of criterion validity, this validity was much greater for the demographically corrected norms than the other two norming methods—43% of PWH were classed as impaired using demographically corrected norms vs. 22.5% using test publisher norms and 19.6% using internal standardization norms.

Regarding between-group differences in the proportion of participants who delivered impaired performance in ≥ 2 cognitive domains (i.e., the proportion displaying Overall Neurocognitive Impairment), analyses detected only one significant finding: When performance was evaluated against the demographically corrected SA norms, 43.1% of PWH (in contrast to 15.8% of controls) were judged as impaired. This contrasts to 22.5% versus for 15.8% test publisher norms, and 19.6% versus 14.0% for internal standardization norms. The implication here, then, is that the demographically corrected norms are more sensitive to impaired performance in PWH, and hence may be more likely to be able to discriminate between individuals with HIV-associated NCI and those without.

One potential concern here, however, is that the demographically corrected norms may overestimate the prevalence of HIV-associated cognitive impairment – recall that more than 40% of PWH were classed as impaired when we used this method, however, we held the domain-impairment rate to a constant 15% in the control group, which alleviates this concern considerably. Furthermore, several studies indicate that, in PWH who are virally suppressed and/or who are stable on ART regimens, this prevalence ranges from 4.76% to 80% (Habib et al. 2013; Sacktor et al. 2016; Saloner & Cysique 2017). Of note is that a recent systematic review and meta-analysis of the prevalence of HIV-associated neurocognitive disorder (HAND; diagnosed based on the Frascati criteria) estimated the number to be closer to 45% (Wei et al. 2020).

Overall, rates of NCI as gauged by GDS (controls = 14.9%, PWH = 37.3%) are more conservative and account better for test correlation within the test battery; however, consideration of the impairment pattern within each cognitive domain when calculating GDS is important as the nuances of how GDS is calculated has implications for HIV-associated NCI and differential diagnosis (Blackstone et al. 2012).

We offer three reasons in support of a preference for demographically corrected SA norms over internal standardization norms when one is seeking accurate detection of HIV-associated NCI.

First, when using demographically corrected SA norms the proportion of controls who fell within the normal distribution more closely matched the expected range of neurocognitive impairment (Mekuriaw et al. 2023; Wang et al. 2020). Second, when evaluated against demographically corrected SA norms PWH performance was significantly more impaired than that of controls in all the major domains typically associated with HIV-associated NCI (Heaton et al. 2010). This was not the case for internal-standardization norms. Third, when the internal standardization norms were used to group participants into cognitively impaired / unimpaired subsets, analyses detected significant-between group differences for age and education in the control sample and for education in the PWH sample. No such differences were detected when the impaired/unimpaired classification was made using demographically corrected SA norms.

As expected, the demographically corrected normative dataset was most effective at adjusting appropriately for demographic effects on neuropsychological test performance, thus allowing interpretation of performance that is largely unclouded by demographic confounds. It must however be kept in mind that reducing demographic associations is not panacea. For example, higher education often results in other benefits such as lower rates of organic impairment due to associated factors such as higher cognitive reserve, higher socioeconomic status, and better health care access. Thus, eliminating education effects could result in over-correction. In contrast to the demographically corrected norms, for most of the measured outcomes test publisher norms did not correct for education and only partially corrected for age. This finding has important implications for both research and clinical practice in low- and middle-income countries such as South Africa, where use of test publisher norms remains frequent. It seems clear that uncritical use of such norms is not appropriate in this LMIC setting (and, by implication, in other LMICs). The other form of norming methodology considered here, internal standardization, is also often used as a basis for clinical interpretation of test performance by a patient or patient group. However, this method does not fully correct for demographic effects, leaving residual effects in the impairment measurement (Freedman & Manly 2015). Regarding reducing demographic associations with test scores, while this is generally good practice.

### Limitations and directions for future research

A few aspects of the study design may limit the generalizability of inferences one might draw from our findings. First, we used self-report to account for the presence or absence of other diseases and disorders (e.g., diabetes mellitus, hypertension) that may have affected neurocognitive test performance, and we did not rule out all possible diseases/disorders. Second, rather than being specifically matched to the PWH group on key sociodemographic variables, the control group was stratified by age, sex, and education. However, we chose this stratification strategy because control samples closely matched to clinical study samples may produce internal standardization norms that are less applicable to the general population. Third, within the internal standardization norms dataset *z*-scores were not stratified according to age, education, and sex because some of the cell sizes were too small to generate meaningful means and standard deviations (variability, particularly, is lost within the small cells). This may have resulted in these norms being less-than-optimally sensitive to impairment. Fourth, interactions among demographic variable (e.g., age*education) are not included in the demographically adjusted norm development. Fith,, although our control and PWH groups were homogenous, we did not collect data on potentially important individual-difference factors such as socioeconomic status (as indexed by, for instance, occupational status, personal and household income, and assets). It is particularly important to include such variables in statistical models when evaluating neuropsychological test performance in countries such as South Africa, where there are large disparities in wealth and economic equality. Sixth, we did not collect data on quality of education, a variable that can have significant impact on neuropsychological test performance (Chin et al. 2012; Manly et al. 2002). However, all participants were drawn from the same community, making it unlikely that there would be large within-sample disparities in quality of education. Seventh, we do not have external validity indicators for the presence of cognitive impairment. Future studies should include a validity indicator, e.g., clinician ratings, longitudinal decline, biomarkers, and/or brain metrics. Finally, because our set of demographically corrected SA norms were developed using a demographically limited data set, it is not known whether they are generalizable to, for instance, other language/ethnic groups within South Africa or demographically similar populations elsewhere in Africa and in LMICs across the world. Nonetheless, we hope the approach we describe here will be valuable to future research and to clinicians in LMICs, especially considering the numerous obstacles to developing normative data in these settings.

## Summary and Conclusion

The need for norms suitable to LMICs and particular subpopulations within HICs is particularly urgent. This study contributes to a growing literature speaking directly to the importance of using culturally appropriate norms for people who do not match the population from which original normative data were drawn, and the importance of valid norms to health disparity research (Kamalyan et al. 2019; Vecchio et al. 2019).

To emphasize the importance of clinicians and researchers carefully considering their choice of tests and norming adjustments when assessing clients from cultures and backgrounds other than the original population from which the norms were drawn, we compared the performance of three different methods of generating normative data (test publisher norms, internal standardization norms, and demographically corrected South African norms) in evaluating the cognitive test scores of a group of healthy South African adults and a matched group of people with HIV. Because more than 7 million PWH are resident in South Africa, and because a relatively large percentage of them will present with concomitant neurocognitive impairment, it is important that the already overburdened South African healthcare system has tools that will allow accurate and efficient detection of such impairment (Avert 2019; Habib et al. 2013; Vecchio et al. 2019).

Our data highlighted the major risks of using test publisher norms (particularly in batteries featuring tests that were not co-normed) in patients or groups different from the standardization sample. Our data also highlighted that using internal standardization norms risked not accounting for effects of potential demographic confounders (e.g., age and education) when interpreting test performance. Importantly, we showed that demographically corrected SA norms can be developed successfully from a relatively small sample of participants (*n* = 114), and that these norms can be used successfully in a LMIC setting to elicit evidence of neurocognitive impairment in PWH.

Using the regression-based norming method that allowed us to derive the demographically corrected SA norms may enable neuropsychologists in LMICs to create population-appropriate norms to inform both research and clinical practice. We suggest that our demographically corrected SA norms are suitable for use in South Africa with patients who match our study sample demographics. An MS Excel spreadsheet that automatically corrects raw performance based on these normative formulas is available from 10.25375/uct.14718192 (please reference this paper when using this resource). Naturally, caution should be taken with interpretation of test scores when applying these demographically corrected SA norms to individuals outside of our stipulated sample. However, in instances where patient demographics are similar to our sample these norms may hold great value, providing guidance on how to interpret test performance when compared to existing norms that are demographically more distant to the patient.

## Supplementary Information

Below is the link to the electronic supplementary material.

Supplementary file1 (PDF 246 KB)
