# Supplementary material for: Generating fair, reliable, and accurate neuropsychological test norms for people with HIV in a low- or middle-income country
Source: J Neurovirol. 2024 Dec 30;31(1):41–55. doi: 10.1007/s13365-024-01235-6 (PMC11971196; doi:10.1007/s13365-024-01235-6)
Supplement: Supplementary file 1 — Supplementary file1 (PDF 246 KB) [file 13365_2024_1235_MOESM1_ESM.pdf]

Supplementary materials

Table S1

Cognitive Domains, Neuropsychological Tests, and Sources of US-publisher Normative Data

Domain / Test	Source of Normative Data	Demographic corrections
Motor Function		
Grooved Pegboard Test	Ruff and Parker (1993), as reported in the GPT User's Manual	Age and gender
Processing Speed		
Trail Making Test Part A[a]	Dodrill (1987)	Age
Color Trails Test 1	D'Elia, Satz, Uchiyama, and White (1996)	Age and education
WAIS-III Symbol Search	Wechsler (1997)	Age
WAIS-III Digit Symbol Coding	Wechsler (1997)	Age
Attention/Working Memory		
WMS-III Spatial Span	Wechsler (1997)	Age
WAIS-III Digit Span	Wechsler (1997)	Age
Language		
Category Fluency		
Animals[a]	Tombaugh, Kozak, and Rees (1999)	Age
Learning		
HVLT-R Total Learning	Brandt and Benedict (2001)	Age
BVMT-R Total Learning	Benedict (1977)	Age
Memory		
HVLT -R Delayed Recall Total	Brandt and Benedict (2001)	Age
BVMT-R Delayed Recall Total	Benedict (1977)	Age
Executive Function		
Color Trails Test 2	D'Elia et al. (1996)	Age and education
WCST (Total Correct)	Kongs, Thompson, Iverson, and Heaton (2000)	Age

Note. GPT = Grooved Pegboard Test; WAIS-III = Wechsler Adult Intelligence Scale, Third Edition; WMS-III = Wechsler Memory Scale, Third Edition; HVLT-R = Hopkins Verbal Learning Test-Revised; BVMT-R = Brief Visuospatial Memory Test-Revised;WCST = Wisconsin Card Sorting Test.

[a]US-publisher norms were used for all tests except these.

Table S2

Neuropsychological Test Performance: Descriptive Statistics and Between-Group Comparisons (N = 216)

	Group				
	Healthy Controls (n = 114)		PWH (n = 102)		
Domain / Outcome Variable	*M*	*SD*	*M*	*SD*	*p*
Motor Function					
Grooved Pegboard Test					
Dominant hand[a]	70.08	25.29	76.87	20.73	**.03***
Nondominant hand[a]	78.89	26.25	88.75	25.56	**.006****
Processing Speed					
TMT-A[a]	40.92	15.85	50.75	19.01	**< .001*****
CTT1[a]	52.45	18.55	58.48	18.56	**.02***
WAIS-III Symbol Search	19.39	7.19	22.09	5.50	**.03***
WAIS-III Digit Symbol	44.76	13.35	40.39	13.45	**.02***
Attention/Working Memory					
WMS-III Spatial Span	10.01	2.51	10.13	2.42	.73
WAIS-III Digit Span	12.12	2.86	10.87	2.25	**< .001*****
Language					
Category Fluency					
Animals	13.82	3.51	13.61	3.05	.64
Fruits and Vegetables	14.02	3.59	14.68	3.14	.16
Learning					
HVLT-R Total Learning	24.71	4.17	23.03	3.48	**.001****
BVMT-R Total Learning	12.96	5.91	12.01	5.80	.24
Memory					
HVLT -R Delayed Recall Total	8.54	2.02	7.47	1.99	**< .001*****
BVMT-R Delayed Recall Total	5.34	2.55	5.24	2.93	.78
Executive Function					
CTT2[a]	117.95	47.75	124.57	47.33	.31
WCST (total correct)[b]	36.11	11.69	36.95	13.41	.62

Note. PWH = People Living with HIV; ESE = effect size estimate (Cohen's *d*); TMT-A = Trail Making Test Part A; CTT1 = Color Trails Test 1; WAIS-III = Wechsler Adult Intelligence Scale, Third Edition; WMS-III = Wechsler Memory Scale, Third Edition; HVLT-R = Hopkins Verbal Learning Test-Revised; BVMT-R = Brief Visuospatial Memory Test-Revised; CTT2 = Color Trails Test 2; WCST = Wisconsin Card Sorting Test.

[a]On these timed tests a higher score indicates poorer performance. [b]HC data based on $n = 112$ (two participant did not complete the test).

*$p < .05$. **$p < .01$. ***$p < .001$. All listed *p*-values are two-tailed. Statistically significant *p*-values are marked in boldface font.

Table S3

Internal standardization norms: Means and standard deviations for neuropsychological test performance in the healthy control sample, stratified by age (N = 114)

Age Range	n	HVLT-R	HVLT-R Delayed Recall	Spatial Span Forward	Spatial Span Backward	Spatial Span	TMTA	CTT1	CTT2	BMVT-R	BMVT-R Delayed Recall	Digit Span Forward	Digit Span Backward
18-25	32	24.88 (4.37)	8.56 (2.21)	6.00 (1.55)	4.84 (1.48)	10.84 (2.59)	33.04 (10.47)	46.27 (14.33)	97.83 (23.67)	16.00 (4.88)	6.41 (2.06)	7.91 (1.78)	4.50 (2.06)
26-35	27	25.19 (4.14)	8.74 (1.58)	5.22 (1.53)	5.37 (1.42)	10.59 (2.68)	34.63 (12.01)	45.64 (12.94)	94.56 (28.46)	14.44 (5.60)	5.96 (2.19)	8.11 (1.37)	4.89 (1.72)
36-45	27	24.48 (4.14)	8.33 (2.08)	5.07 (1.30)	4.70 (1.10)	9.78 (1.97)	43.92 (14.22)	51.91 (16.23)	126.89 (42.80)	11.63 (6.20)	5.00 (2.91)	7.56 (1.87)	4.15 (1.13)
46-55	25	24.40 (4.13)	8.36 (1.98)	4.80 (1.80)	4.08 (1.22)	8.88 (2.26)	53.34 (18.45)	65.75 (22.59)	141.01 (60.67)	9.60 (4.79)	3.92 (2.27)	7.44 (1.61)	4.16 (1.18)

Age Range	n	Digit Span	Digit Symbol	Symbol Search	Animals	Fruit & Vegetables	Grooved Pegboard DH	Grooved Pegboard NDH	WCST Total Correct	WCST Categorie s
18-25	32	12.41 (3.43)	54.06 (10.91)	23.16 (6.04)	13.38 (3.68)	13.78 (2.80)	63.66 (9.65)	73.361 (2.26)	39.74 (10.35)	2.42 (1.15)
26-35	27	13.00 (2.50)	48.59 (10.77)	23.11 (5.73)	15.07 (2.97)	15.15 (3.68)	63.161 (2.52)	73.181 (3.48)	35.85 (12.91)	1.92 (1.38)
36-45	27	11.70 (2.73)	42.67 (10.77)	16.78 (7.21)	13.22 (3.50)	13.30 (4.17)	72.461 (4.76)	75.881 (8.94)	34.11 (11.15)	1.70 (1.27)

46-55	25	11.60 (2.38)	32.60 (10.07)	14.68 (4.63)	13.76 (3.76)	13.88 (3.81)	73.811 (2.41)	85.871 (7.08)	9.18 (2.19)	9.33 (3.00)	34.52 (12.33)	1.60 (1.29)

Note. HVLT-R = Hopkins Verbal Learning Test-Revised; TMT-A = Trail Making Test Part A; CTT1 = Color Trails Test 1; CTT2 = Color Trails Test 2; BVMT-R = Brief Visuospatial Memory Test-Revised; WCST = Wisconsin Card Sorting Test; DH = Dominant Hand; NDH = Non-dominant Hand Digit Span, Digit Symbol and Symbol Search are from the Wechsler Adult Intelligence Scale, Third Edition; Spatial Span is from the Wechsler Memory Scale, Third Edition.

Table S4.1

Regression Analyses: Final Models for Tests of Motor Function (N = 216)

Test / Outcome Variable	Predictor	*B*	SE *B*	*t*	*p*	*ß*	R^2
Grooved Pegboard Test							
Dominant hand	Constant	7.50	2.27	3.31**	.001		
	Age	-0.09	0.02	-3.94***	<.001	-.35	
	Age^2	0	0.002	0.05	.957	.005	
	Education	0.59	0.19	3.16**	.002	.29	
	$Education^2$	-0.04	0.14	-0.32	.751	-.03	
	Gender	-1.08	0.48	-2.23*	.028	-.19	.23
Nondominant hand	Constant	8.21	2.31	3.55***	<.001		
	Age	-0.07	0.02	-3.07**	.003	-.28	
	Age^2	-0.002	0.002	-0.89	.374	-.08	
	Education	0.46	0.19	2.42*	.017	.23	
	$Education^2$	-0.10	0.14	-0.73	.468	-.07	
	Gender	-0.36	0.49	-0.73	.469	-.06	.18

Note. $^*p < .05$. $^{**}p < .01$. $^{***}p < .001$.

Table S4.2

Regression Analyses: Final Models for Tests of Processing Speed (N = 216)

Test / Outcome Variable	Predictor	*B*	SE *B*	*t*	*p*	*ß*	R^2
Trail Making Test Part A							
	Constant	8.22	2.06	3.98***	<.001		
	Age	-0.12	0.02	-6.01***	<.001	-.50	
	Age^2	0.002	0.002	1.02	.310	.09	
	Education	0.55	0.17	3.28**	.001	.28	
	$Education^2$	0.01	0.13	0.08	.939	.007	
	Gender	-0.30	0.44	-0.68	.499	-.05	0.32
Color Trails Test 1	Constant	6.53	2.29	2.85**	.005		
	Age	-0.08	0.02	-3.42***	<.001	-.30	
	Age^2	-0.002	0.002	-1.32	.191	-.12	
	Education	0.63	0.19	3.33**	.001	.30	
	$Education^2$	0.02	0.14	0.12	.907	.01	
	Gender	-0.35	0.49	-0.70	.483	-.06	0.23
WAIS-III Symbol Search	Constant	8.91	1.99	4.49***	<.001		
	Age	-0.12	0.02	-6.18***	<.001	-.50	
	Age^2	0.001	0.002	0.81	.419	.07	
	Education	0.48	0.16	2.93**	.004	.25	
	$Education^2$	-0.10	0.12	-0.80	.426	-.07	
	Gender	0.30	0.42	0.71	.477	.05	0.36
WAIS-III Digit Symbol Coding	Constant	6.86	1.85	3.70***	<.001		
	Age	-0.15	0.02	-8.25***	<.001	-.61	

Age^2	0.002	0.002	1.35	.180	.10	
Education	0.74	0.15	4.86***	<.001	.37	
Education^2	0.19	0.11	1.67	.098	.13	
Gender	-0.37	0.40	-0.93	.355	-.06	0.47

Note. WAIS-III = Wechsler Adult Intelligence Scale, Third Edition.
*p < .05.**p < .01. *** p < .001.

Table S4.3

Regression Analyses: Final Models for Tests of Attention and Working Memory (N = 216)

Test / Outcome Variable	Predictor	*B*	SE *B*	*t*	*p*	*ß*	R^2
WMS-III Spatial Span	Constant	8.44	2.44	3.46***	<.001		
	Age	-0.07	0.02	-3.05**	.003	-.29	
	Age^2	0	0.002	-0.20	.842	-.02	
	Education	0.32	0.20	1.59	.115	.15	
	$Education^2$	0.07	0.15	0.50	.620	.05	
	Gender	1.26	0.52	2.42*	.017	.21	.14
WAIS-III Digit Span	Constant	4.80	2.30	2.09*	.039		
	Age	-0.04	0.02	-1.74	.084	-.17	
	Age^2	0.002	0.002	1.01	.316	.01	
	Education	0.62	0.19	3.31**	.001	.33	
	$Education^2$	-0.02	0.14	-0.12	.905	-.01	
	Gender	-0.46	0.49	-0.94	.348	-.09	.10

Note. WMS-III = Wechsler Memory Scale, Third Edition; WAIS-III = Wechsler Adult Intelligence Scale, Third Edition.
$*p < .05$. $**p < .01$. $***p < .001$.

Table S4.4

Regression Analyses: Final Models for Tests of Language (N = 216)

Category Fluency Test	Predictor	*B*	SE *B*	*t*	*p*	*ß*	R^2
Animals	Constant	3.81	2.35	1.62	.108		
	Age	0	0.02	0.02	.984	.002	
	Age^2	0	0.002	-0.08	.934	-.01	
	Education	0.52	0.19	2.73**	.008	.28	
	$Education^2$	0.25	0.14	1.74	.085	.18	
	Gender	0.51	0.5	1.02	.310	.10	.04
Fruits and Vegetables	Constant	4.60	2.31	1.99*	.049		
	Age	-0.01	0.02	-0.46	.648	-.04	
	Age^2	0	0.002	-0.10	.918	-.01	
	Education	0.61	0.19	3.23**	.002	.30	
	$Education^2$	0.14	0.14	0.97	.333	.09	
	Gender	-2.38	0.49	-4.83***	<.001	-.41	.19

Note. *$p < .05$.**$p < .01$. *** $p < .001$.

Table S4.5

Regression Analyses: Final Models for Tests of Learning (N = 216)

Test / Outcome Variable	Predictor	B	SE B	t	p	$ß$	R^2
HVLT-R Total Learning	Constant	7.01	2.55	2.75**	.007		
	Age	-0.01	0.02	-0.57	.570	-.06	
	Age^2	0.002	0.002	0.81	.419	.08	
	Education	0.42	0.21	2.02*	.046	.20	
	Education^2	-0.08	0.15	-0.54	.592	-.05	
	Gender	-1.90	0.54	-3.49***	<.001	-.32	.01
BVMT-R Total Learning	Constant	8.09	2.29	3.54***	<.001		
	Age	-0.11	0.02	-4.84***	<.001	-.43	
	Age^2	0.001	0.002	0.60	.548	.06	
	Education	0.50	0.19	2.67**	.009	.25	
	Education^2	0.12	0.14	0.89	.378	.08	
	Gender	-0.01	0.49	-0.02	.985	-.002	.21

Note. HVLT-R = Hopkins Verbal Learning Test-Revised; BVMT-R = Brief Visuospatial Memory Test-Revised.
*p < .05.**p < .01. *** p < .001.

Table S4.6

Regression Analyses: Final Models for Tests of Memory (N = 216)

Test / Outcome Variable	Predictor	*B*	SE *B*	*t*	*p*	*ß*	R^2
HVLT-R Delayed Recall	Constant	3.36	2.45	1.37	.173		
	Age	-0.01	0.02	-0.24	.813	-.02	
	Age^2	0.002	0.002	0.90	.370	.09	
	Education	0.65	0.20	3.25**	.002	.32	
	$Education^2$	0.21	0.15	1.41	.160	.14	
	Gender	-1.27	0.52	-2.44*	.016	-.22	.09
BVMT-R Delayed Recall	Constant	10.63	2.31	4.60***	<.001		
	Age	-0.08	0.02	-3.75***	<.001	-.36	
	Age^2	0	0.002	0.05	.962	.005	
	Education	0.21	0.19	1.12	.265	.11	
	$Education^2$	0.16	0.14	1.15	.255	.12	
	Gender	-0.57	0.49	-1.16	.248	-.10	.10

Note. HVLT-R = Hopkins Verbal Learning Test-Revised; BVMT-R = Brief Visuospatial Memory Test-Revised.
$^*p < .05$. $^{**}p < .01$. $^{***}p < .001$.

Table S4.7

Regression Analyses: Final Models for Tests of Executive Function (N = 216)

Test / Outcome Variable	Predictor	*B*	SE *B*	*t*	*p*	*ß*	R^2
Color Trails Test 2	Constant	7.01	2.18	3.21**	.002		
	Age	-0.11	0.02	-5.11***	<.001	-.44	
	Age^2	0	0.002	-0.21	.834	-.02	
	Education	0.61	0.18	3.43***	<.001	.30	
	$Education^2$	0.16	0.13	1.25	.215	.11	
	Gender	0.05	0.47	0.12	.909	.01	.28
WCST Total Correct	Constant	7.65	2.49	3.08**	.003		
	Age	-0.05	0.02	-1.96	.053	-.19	
	Age^2	0.001	0.002	0.61	.545	.06	
	Education	0.41	0.20	2.03*	.045	.21	
	$Education^2$	-0.03	0.15	-0.19	.851	-.02	
	Gender	-0.95	0.53	-1.79	.077	-.17	.06

Note. WCST = Wisconsin Card Sorting Test.

*p < .05.**p < .01. *** p < .001.
